# Framework for implementing collaborative TB-silicosis activities in India: insights from an expert panel

**DOI:** 10.1186/s13690-024-01325-1

**Published:** 2024-06-18

**Authors:** Mihir P. Rupani, Pankaj Nimavat, Yogesh Patel, Harsh D. Shah, Arkaprabha Sau

**Affiliations:** 1 Clinical Epidemiology (Division of Health Sciences), ICMR - National Institute of Occupational Health (NIOH), Indian Council of Medical Research (ICMR), Meghaninagar, Ahmedabad City, Gujarat 380016 India; 2 State Tuberculosis Training and Demonstration Center (STDC), Civil Hospital, Asarwa, Ahmedabad City, Gujarat 380016 India; 3 John Snow India (JSI) Private Limited, New Delhi, 110070 India; 4 Indian Institute of Public Health (IIPH), Palaj Village, Gandhinagar, 382042 Gujarat India; 5 Deputy Director (Medical), Regional Labour Institute, Directorate General Factory Advice Service & Labour Institutes, Kanpur, Uttar Pradesh 208005 India

**Keywords:** Silico-tuberculosis, Integration, National tuberculosis elimination program, Joint activities, Silica dust, Program guidelines, Collaborative framework, Bidirectional activities, India

## Abstract

Tuberculosis (TB) treatment is more challenging for patients with silicosis, as it complicates the diagnosis of both diseases and increases mortality risk. Silicosis, an incurable occupational disease, confounds the diagnosis of TB and vice versa, making it more difficult to accurately identify and treat either condition. Moreover, TB appears to accelerate the progression of silicosis. Exposure to silica dust, a common cause of silicosis, can also trigger latent TB to become active TB. This correspondence outlines a proposed framework for implementing collaborative TB-silicosis activities in India, aimed at improving early diagnosis and management for both diseases. An expert panel of medical professionals developed this framework through online consultations in October and November 2022. The panel's goal was to establish a consensus on integrating TB-silicosis activities, with a focus on early detection and proper management. The framework suggests testing all patients with silicosis for active TB and screening workers exposed to silica dust for latent TB infection. It also recommends that patients with TB who have a history of occupational exposure to silica dust should be tested for silicosis. Reliable diagnostic tools, such as chest X-rays, are emphasized, providing guidance on their use for both diseases. The proposed collaborative TB-silicosis framework offers a structured approach to identifying and managing these two diseases, contributing to the global goal of eliminating silicosis by 2030 and aligning with the World Health Organization’s targets for reducing TB incidence and mortality. It recommends specific strategies for implementation, including testing, referral systems, and workplace-based interventions. The framework also underscores the need for coordinated efforts among stakeholders, including the ministries of health, labor, industry, and environment. This correspondence provides valuable insights into how India can successfully implement collaborative TB-silicosis activities, serving as a model for other regions with similar challenges.

**Table:** 

**Text box 1. Contributions to the literature**
• This correspondence addresses the dual challenge of tuberculosis (TB) and silicosis in India, presenting a collaborative framework for early diagnosis and treatment. • It proposes workplace-based interventions and cost-effective diagnostic strategies, which can be valuable for regions with high silica dust exposure. • The paper emphasizes the importance of integrating public health efforts across multiple sectors, providing practical steps to improve healthcare infrastructure and reduce costs. • By drawing insights from an expert panel, the correspondence adds to the discussion on effective TB and silicosis management, offering a model for other countries aiming to address similar occupational health issues.

## Introduction

India has the highest number of tuberculosis (TB) cases in the world, with 2.9 million cases reported in 2022, contributing approximately 27% of the global TB burden of 10.6 million [1, 2]. In contrast, silicosis, a serious occupational illness, had 2.65 million recorded cases globally in 2019, but this figure likely underestimates the actual prevalence due to widespread underreporting [3–8]. Silicosis accounts for 39% of all pneumoconiosis cases and 0.65 million disability-adjusted life years (DALYs), emphasizing its significant impact on public health [9–11]. Countries such as China, South Africa, and Brazil have similar epidemiological profiles for these diseases, highlighting the global relevance of addressing TB and silicosis [12, 13].

The World Health Organization (WHO) aims to eliminate silicosis and TB by 2030, with India targeting TB elimination by 2025 [14–16]. Given that silica dust exposure not only leads to silicosis but also increases the risk of TB, even in the absence of silicosis, a collaborative approach to tackling both diseases has been suggested [10, 17–23]. TB increases the risk of death among patients with silicosis [24–26]. Silicosis, in turn, exacerbates TB treatment outcomes by increasing the risk of death, relapse, treatment failure, and interruptions [27–29]. Additionally, the coexistence of TB and silicosis poses considerable diagnostic challenges due to the difficulty in distinguishing their clinical and radiological presentations [30]. Silicosis is often underdiagnosed if TB is present, and TB is similarly overlooked when silicosis is present [30]. These links underline the critical need for integrated TB-silicosis activities.

A collaborative approach between TB and silicosis control has been highlighted as a way to improve early diagnosis and management [28, 31–35]. In South Africa, efforts to reduce TB in the mining sector have been successfully implemented, recognizing TB as an occupational disease in mining work and emphasizing the reduction of silica dust exposure as a primary preventive measure for all silica-related diseases [36, 37]. For instance, Churchyard's extensive research, including the Thibela study, demonstrated the challenges and outcomes of mass screening and treatment for TB among gold miners [38]. The study found that while isoniazid preventive therapy was effective during treatment, it did not have a long-term impact on TB incidence or prevalence, underscoring the critical need for primary prevention measures such as reducing silica dust exposure [38].

This integration can follow the successful models of existing joint activities in TB-HIV, TB-diabetes, TB-tobacco, and TB-pregnancy initiatives [39–46]. The TB-HIV bidirectional screening program in India, for example, has been particularly effective, highlighting the potential benefits of integrated approaches [47]. Additionally, WHO recommends screening for TB in individuals with current or past exposure to silica dust and providing TB preventive treatment for those diagnosed with silicosis [46, 48, 49]. Given the strong association between TB and silicosis, this correspondence proposes a framework for implementing joint TB-silicosis activities in India. The objective is to establish mechanisms that facilitate screening, diagnosis, health promotion, preventive treatment, rehabilitation, referral, capacity building, awareness generation, reporting, and involvement of private providers and non-governmental organizations.

## Diagnostic strategy and tests for TB and silicosis

### Diagnostic strategy

#### Investigate all silicosis/silica dust exposed individuals for TB

Silica dust exposure weakens the immune system, increasing the risk of TB [17–20, 23]. Additionally, silicosis itself predisposes individuals to TB [10]. Thus, all patients with silicosis and workers exposed to silica dust who exhibit any TB symptoms, regardless of duration, should be tested for TB.

#### Investigate all TB with occupational history for silicosis 

TB patients with a history of occupational exposure to silica dust in industries should be tested for silicosis [28]. Thorough collection of occupational history can help identify individuals at risk for silicosis. However, the presence of TB can confound the diagnosis of silicosis on chest X-rays [30].

#### Bi-directional screening in high silicosis-burden areas

Bi-directional screening in areas with a high burden of silicosis, such as industrial zones, can lead to earlier identification of both silicosis and TB. Since these areas are identified by the National TB Elimination Program (NTEP), active case-finding efforts during TB-silicosis activities can yield significant results.

### Diagnostic tests

#### Chest X-ray as a common diagnostic tool

Chest X-rays can be used to diagnose both TB and silicosis, with digital X-rays providing higher resolution [28, 50]. High-resolution computed tomography (HRCT) is recommended when there's uncertainty in diagnosis [30]. However, HRCT requires specific scanning protocols and the training of radiologists to accurately interpret the results [7, 51, 52]. Additionally, knowledge of classification systems is essential, and there is a risk of false positives that may lead to invasive interventions [51, 52]. A history of silica dust exposure, combined with radiological evidence, can be key in making a more accurate diagnosis.

#### TB among patients with silicosis

India's decentralized system of TB care allows for efficient diagnosis through a network of peripheral health institutions (PHIs) including public health facilities, corporate sector, and faith-based hospitals [53]. This system offers various diagnostic tools, including microscopy, chest X-rays, rapid molecular testing, and culture-sensitivity [53]. Chest X-ray and nucleic acid amplification testing (NAAT) are recommended for diagnosing TB among patients with silicosis [48]. Silicosis is now considered a key population for TB screening [48].

#### Silicosis among patients with TB

A high-resolution CT scan is often considered the gold standard for diagnosing silicosis among TB patients, though it is costly [7]. Most cases of silicosis are distinguishable from TB with high-resolution CT. [7, 10] To assess lung capacity, pulmonary function tests with a spirometer are also useful [10]. Chest X-rays are a more affordable option for diagnosing silicosis [28]. For conclusive diagnosis, radiological findings suggestive of silicosis should be accompanied by an occupational history of silica dust exposure.

## Framework development

To develop a framework for collaborative tuberculosis-silicosis (TB-silicosis) activities in India, the authors themselves formed an expert-panel. This approach brought together medical professionals with diverse expertise, focusing on occupational environments where silica dust is generated, such as quarries, foundries, mining activities, ceramic, cement, and building construction industries [10]. The aim was to formulate collaborative activities between the National TB Elimination Program (NTEP) and the sites where patients with silicosis are likely to be diagnosed, including factories, district and medical college hospitals located near these industries, and Employees State Insurance Corporation (ESIC) dispensaries and hospitals.

The panel consisted of MPR, YP, HDS, and AS who are MDs in Community Medicine, and PN, a medical doctor with a diploma in public health. MPR works at a premier institute on occupational health and has expertise in silicosis and silico-tuberculosis. AS is involved in policymaking related to occupational health in India. PN, YP, and HDS have extensive experience working on TB program implementation in India. Additionally, PN has a keen interest and expertise in silicosis and silico-tuberculosis. MPR, PN, and HDS are based in Gujarat, whereas YP and AS are located in New Delhi and Uttar Pradesh, respectively.

The expert panel met online several times between October and November 2022 to draft an initial policy document for integrating TB and silicosis control. Through these consultations, the panel sought to reach a consensus on the algorithms for implementing collaborative TB-silicosis activities, focusing on early diagnosis, reducing silica exposure, and prompt management of TB in silicosis patients.

The panel's discussions were informed by qualitative findings and existing guidelines for TB and silicosis management [28]. The final framework outlined algorithms for identifying and treating patients with silicosis who may also have TB, as well as those with TB who might be at risk of silicosis due to occupational exposure to silica dust. The collaborative framework aims to improve screening, diagnosis, health promotion, preventive treatment, rehabilitation, referral, capacity building, awareness generation, reporting, and engagement with private providers and non-governmental organizations.

## TB-silicosis collaborative framework

### Testing and management of silicosis among patients with TB

In India, chest X-rays have been proposed as the preferred diagnostic tool for bi-directional TB-silicosis screening activities. When a patient is diagnosed with TB, they are typically counseled on the importance of proper nutrition, the benefits of completing treatment, managing comorbidities, and addressing any addictive behaviors. As part of the collaborative TB-silicosis framework, healthcare workers should also inquire about a patient's occupational history of silica dust exposure using a structured questionnaire (Table 1).

**Table 1:** Questions for eliciting occupational history of silica dust exposure in TB patients

No	Question	Response
1	Have you ever been exposed to dust at your workplace?	Yes / No
1a	If yes, which of the following best describes your current or past job?	
	1) Construction or road-laying activities	Yes / No
	2) Mining, excavation, tunneling, or other underground activities	Yes / No
	3) Stone-related work (e.g., polishing, crushing, grinding, sculpting, slate cutting)	Yes / No
	4) Ceramic-related work (e.g., marble, tiles, bricks)	Yes / No
	5) Pottery, casting (e.g., moulders, sand-blasters)	Yes / No
	6) Manufacturing involving silica (e.g., glass, cement, steel)	Yes / No
1b	If yes to any of the above, what was the duration of this job (in years)?	________
	Were you provided with any dust protection (e.g., masks, ventilation systems)?	Yes / No
2	Have you been diagnosed with any respiratory conditions due to your occupation?	Yes / No
	If yes, please specify:	________

**Fig. 1:**
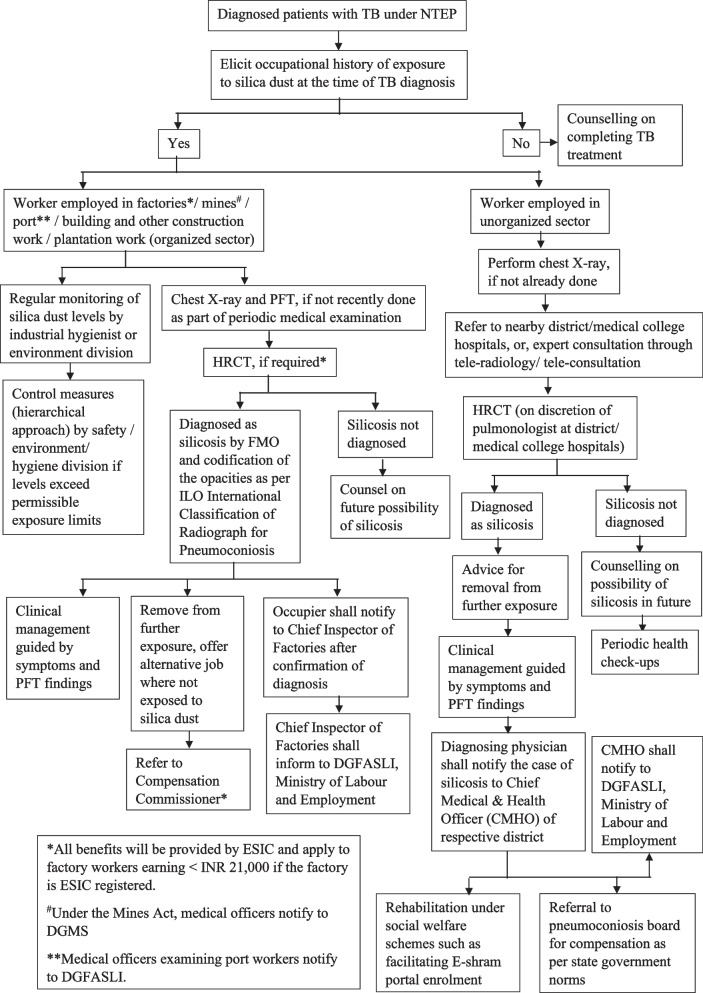
Algorithm for diagnosing silicosis among patients with TB occupationally exposed to silica dust

For workers in the unorganized sector, if a chest X-ray was not obtained during the initial diagnosis of TB, all patients with a history of occupational exposure to silica dust should have one performed (Fig. 1). Referral to nearby district or medical college hospitals, or expert consultation through tele-radiology or tele-consultation platforms like eSanjeevani (https://esanjeevani.in/), may be necessary. TB health staff should also use these opportunities to counsel patients about the potential for co-existing diseases with similar symptoms and emphasize that TB is curable with proper treatment. Workers diagnosed with silicosis should be advised to avoid future exposure to silica dust and seek appropriate rehabilitation resources.

For a more definitive diagnosis of silicosis, high-resolution computed tomography (HRCT) may be required. Workers employed in factories, mines, ports, building and construction work, or plantation work in the organized sector are at risk of exposure to silica dust. Regular monitoring of silica dust levels should be conducted by industrial hygienists or the environment division of the respective industry. Control measures, including a hierarchical approach to managing silica dust levels if they exceed permissible exposure limits, should be implemented by safety, environment, or hygiene divisions [54–56].

For workers in the organized sector, diagnosed cases of silicosis should be confirmed by the Factory Medical Officer (FMO) and opacities codified as per the ILO International Classification of Radiographs for Pneumoconioses [57]. Workers diagnosed with silicosis should be removed from further exposure to silica dust and offered alternative jobs in non-exposure areas. If the factory is registered with the Employees State Insurance Corporation (ESIC), the FMO can refer workers to an ESIC hospital for benefits including medical care, sickness compensation, and rehabilitation [58]. Counseling on the potential future development of silicosis is essential for workers who are exposed to silica dust but have not yet been diagnosed with the disease.

Notifications about cases of silicosis should be sent to the Directorate General Factory Advice Service & Labour Institutes (DGFASLI) and the Directorate General of Mines Safety (DGMS). The Chief Inspectors of Factories (for factories), chief medical & health officers (for the unorganized sector), and medical officers (for mines and ports) should take appropriate actions based on these notifications. The chief medical & health officer (CMHO) of the respective district should facilitate enrolment in social welfare schemes such as the E-shram portal, which ensures unorganized workers receive social security benefits and support during emergencies. Periodic health check-ups and clinical management guided by symptoms and pulmonary function test (PFT) findings are crucial for the ongoing care of workers with silicosis.

### Testing and management of TB among patients with silicosis

Patients with silicosis in India are often diagnosed in factories, at government health facilities and medical college hospitals located near factories, or at Employees State Insurance Corporation (ESIC) dispensaries and hospitals. ESIC is a social security program of the Government of India that provides medical, sickness, and disability benefits to workers in the organized sector through a network of dispensaries and hospitals [58]. Additionally, the Factories Act of 1948 and the Mines Act of 1952 mandate factory medical officers (FMO) in India to conduct periodic medical examinations, including checks for occupational diseases like silicosis. Thus, workers may present to ESIC hospitals, government health facilities, or medical college hospitals with symptoms indicative of silicosis.

**Fig. 2:**
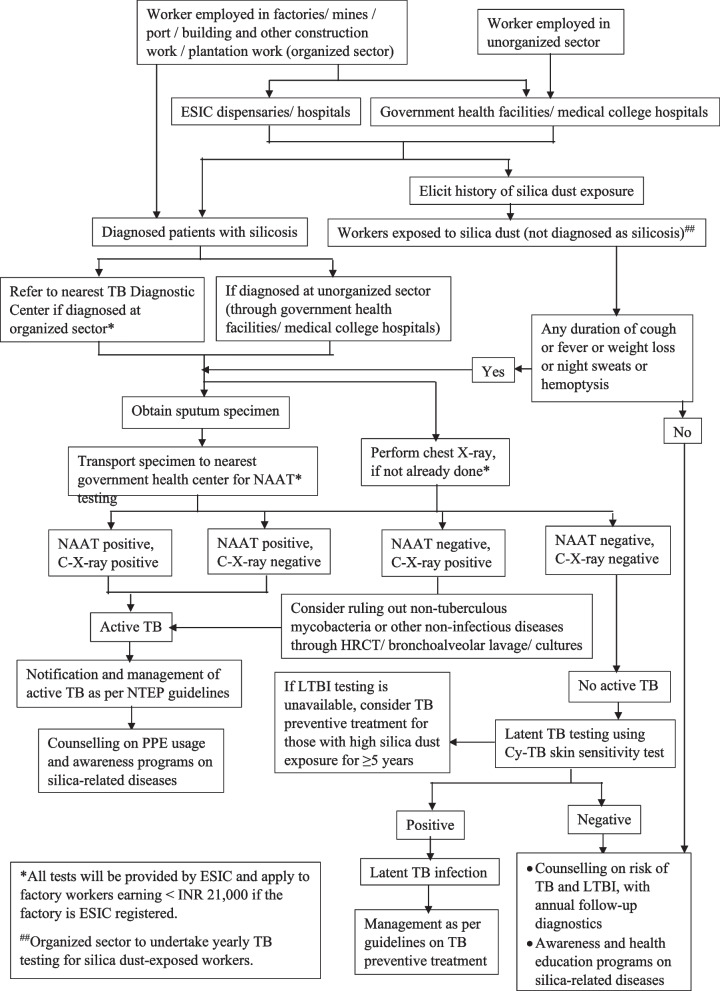
Algorithm for diagnosing TB among patients with silicosis or workers exposed to silica dust

To implement a joint TB-silicosis framework, all patients with silicosis should be tested for active TB disease or infection, regardless of symptoms (Fig. 2). A symptom screen should be conducted on workers with a history of occupational exposure to silica dust, with a positive screen defined by the presence of any duration of cough, fever, weight loss, night sweats, or hemoptysis. If the symptom screen is positive, the worker should undergo all tests specified in the protocol for patients with silicosis.

For patients or workers with a positive symptom screen, a sputum specimen should be collected and transported to the nearest government health clinic for nucleic acid amplification testing (NAAT). In addition to NAAT, a chest X-ray should be obtained concurrently if not already done during the diagnosis of silicosis. Workers exposed to silica dust but not diagnosed with silicosis should also undergo chest X-ray screening. If either the NAAT or chest X-ray results are positive, the patient or worker is diagnosed with active TB and should be treated according to national TB program guidelines. If chest X-ray results are positive, however NAAT results are negative, further testing should be conducted to rule out non-tuberculous mycobacteria or other non-infectious diseases through high-resolution computed tomography (HRCT), bronchoalveolar lavage, or cultures. If these conditions are ruled out, then further management for active TB should be initiated as per national TB program guidelines.

If both chest X-ray and NAAT tests are negative, further assessment for latent TB infection (LTBI) should be conducted using low-cost Cy-TB skin sensitivity tests [59, 60]. If LTBI testing is unavailable, TB preventive treatment should be considered for those with high silica dust exposure for durations of five years or more [61]. Management of latent TB infection should follow the TB preventive treatment guidelines.

It is crucial to counsel patients with silicosis about the possibility of having two co-existing diseases with overlapping symptoms. They should also be informed that, while TB is treatable, silicosis has no cure. Workers diagnosed with silicosis should be advised to avoid further exposure to silica dust to prevent further complications.

Organized sector workers should undergo yearly testing for active TB disease as part of the periodic medical examination. All workers, especially those in the organized sector, should be counseled on personal protective equipment (PPE) usage, and awareness programs on silica-related diseases should be conducted. Patients with active TB should be notified and managed according to NTEP guidelines, and regular follow-ups should be conducted to ensure adherence to treatment protocols.

## Consideration of other silica-related diseases and comorbidities

In addition to silicosis and tuberculosis, other silica-related diseases such as chronic obstructive pulmonary disease (COPD), autoimmune diseases, and lung cancer must be considered. Medical doctors treating patients with these diseases should be vigilant for the presence of TB, as symptoms may overlap with the underlying disease. Special attention should be given to patients with autoimmune connective tissue diseases who are treated with immunosuppressants, corticosteroids, and immunobiologicals, as these medications increase the risk of TB. Therefore, it is proposed that countries incorporate protocols addressing the presence of these comorbidities based on their needs and the availability of tests to ensure comprehensive care.

## Challenges in implementation

Despite the significant health risks associated with silicosis, the disease often receives less attention compared to TB, which benefits from international support from organizations like the WHO and international donors. This discrepancy can be attributed to several factors. Silicosis is generally considered incurable, with a focus on prevention rather than treatment, making it a lower priority for health departments. Additionally, addressing the root causes of silicosis, such as silica dust control and worker safety, requires significant investment and regulatory oversight, which can impact the livelihoods of low-income workers. This challenge is compounded by the complexities of workers' compensation and legal responsibilities, often seen as outside the direct scope of TB programs or health departments.

The Employees State Insurance Corporation (ESIC) provides compensation and medical benefits to workers diagnosed with silicosis, but these benefits are generally available only to those with regular employment contracts. This limitation leaves a significant portion of the workforce unprotected, particularly informal and temporary workers who are often at greater risk of exposure to silica dust. A major challenge in addressing silicosis is tracking workers across companies and after retirement, as TB and silicosis often become apparent only after a worker has exited employment. This issue requires robust legislation and the establishment of a national database to ensure continuous monitoring and healthcare provision for workers, regardless of their employment status. Implementing such a system would facilitate early diagnosis and management of silicosis and TB, even after workers have retired or moved between jobs.

## Recommendations for implementation

### Training modules

Develop a national joint TB-silicosis framework that aligns with the National Strategic Plan 2017–2025 [16]. Create training modules for healthcare personnel and a cascade training program, starting at the state level and moving to peripheral health workers. Consider using online platforms or hybrid approaches for training. Emphasize the importance of gathering occupational history of silica dust exposure. Additionally, include training for radiologists and medical officers to read X-rays for silicosis using standard ILO radiographs for pneumoconioses.

### Awareness generation

Distribute Information, Education, and Communication (IEC) materials on occupational dust exposure risks to factory workers. Tailor awareness campaigns to silicosis patients and workers in silica-producing industries. Use diverse methods such as social media, local television ads, radio jingles, posters, and banners. National hotline numbers can further spread awareness about TB symptoms, risk factors, and when to seek medical attention.

### Maintaining records and reporting

Encourage periodic exchange of line lists of suspected TB-silicosis cases with counterpart organizations for effective coordination. Update TB treatment cards and registers to include detailed occupational history. Ensure this information is reflected on the app and web-based interface, such as Nikshay. Government health centers should track and report the number of silicosis patients screened for TB.

### Monitoring indicators

Establish indicators to track progress in the national TB program and government health centers (refer to Table 2 and Table 3 for specific indicators). These indicators should measure progress in testing, treatment, and completion rates for both active TB disease and latent TB infection among silicosis patients and silica-exposed workers.

**Table 2:** Indicators for monitoring progress toward screening for silicosis for TB patients with occupational silica dust exposure

Indicator	Numerator	Denominator
Proportion of registered TB patients with an occupational history of silica dust exposure	Number of TB patients with an occupational history of silica dust exposure during the reporting period	Total number of TB patients registered during the reporting period
Proportion of TB patients with an occupational history of silica dust exposure receiving counseling on silicosis	Number of TB patients with an occupational history of silica dust exposure who received counseling on silicosis during the reporting period	Number of TB patients with an occupational history of silica dust exposure during the reporting period
Proportion of TB patients with occupational history of silica dust exposure screened for silicosis	Number of TB patients with an occupational history of silica dust exposure who were screened for silicosis during the reporting period	Number of TB patients with an occupational history of silica dust exposure receiving counseling on silicosis during the reporting period
Proportion of TB patients with an occupational history of silica dust exposure receiving high-resolution computed tomography (HRCT) for silicosis diagnosis	Number of TB patients with an occupational history of silica dust exposure who received HRCT for silicosis diagnosis during the reporting period	Number of TB patients with an occupational history of silica dust exposure screened for silicosis during the reporting period
Proportion of screened TB patients diagnosed with silicosis	Number of TB patients diagnosed with silicosis during the reporting period	Total number of TB patients with an occupational history of silica dust exposure screened for silicosis during the reporting period
Proportion of TB patients referred for management of silicosis	Number of TB patients referred for silicosis management during the reporting period	Total number of TB patients diagnosed with silicosis during the reporting period
Proportion of TB patients with silicosis who received symptomatic management	Number of TB patients referred for silicosis who received symptomatic management during the reporting period	Total number of TB patients referred for silicosis management during the reporting period

**Table 3:** Indicators for monitoring progress toward testing for TB among patients with silicosis or workers exposed to silica dust

Indicator	Numerator	Denominator
Proportion of workers counseled on the hazards of silica dust exposure	Number of workers counseled on the hazards of silica dust exposure during the reporting period	Total number of workers employed in silica-dust-exposed industries during the reporting period
Proportion of workers tested for silicosis	Number of workers tested for silicosis during the reporting period	Total number of workers counseled on the hazards of silica dust exposure during the reporting period
Proportion of workers diagnosed with silicosis	Number of workers diagnosed with silicosis during the reporting period	Total number of workers tested for silicosis during the reporting period
Proportion of workers exposed to silica dust symptomatic for active TB	Number of workers exposed to silica dust with any of cough, fever, weight loss, night sweats, or hemoptysis during the reporting period	Total number of workers exposed to silica dust during the reporting period
Proportion of patients with silicosis or workers exposed to silica dust tested for active TB	Number of patients with silicosis or workers exposed to silica dust tested for active TB during the reporting period	Number of patients with silicosis or workers exposed to silica dust symptomatic for active TB during the reporting period
Proportion of patients with silicosis or workers exposed to silica dust found positive for active TB	Number of patients with silicosis or workers exposed to silica dust found positive for active TB during the reporting period	Number of patients with silicosis or workers exposed to silica dust tested for active TB during the reporting period
Proportion of patients with silicosis or workers exposed to silica dust put on TB treatment	Number of patients with silicosis or workers exposed to silica dust put on TB treatment during the reporting period	Number of patients with silicosis or workers exposed to silica dust found positive for active TB during the reporting period
Proportion of patients with silicosis or workers exposed to silica dust who completed TB treatment	Number of patients with silicosis or workers exposed to silica dust who completed the full course of TB treatment during the reporting period	Number of patients with silicosis or workers exposed to silica dust put on TB treatment during the reporting period
Proportion of patients with silicosis or workers exposed to silica dust tested for latent TB infection (LTBI)	Number of patients with silicosis or workers exposed to silica dust tested for latent TB infection during the reporting period (after testing negative for active TB)	Total number of patients with silicosis or workers exposed to silica dust testing negative for active TB during the reporting period
Proportion of patients with silicosis or workers exposed to silica dust put on TB preventive treatment (TPT)	Number of patients with silicosis or workers exposed to silica dust put on TB preventive treatment during the reporting period (after testing positive for LTBI)	Total number of patients with silicosis or workers exposed to silica dust tested positive for LTBI during the reporting period
Proportion of patients with silicosis or workers exposed to silica dust who completed the full course of TB preventive treatment (TPT)	Number of patients with silicosis or workers exposed to silica dust who completed the full course of TB preventive treatment during the reporting period	Total number of patients with silicosis or workers exposed to silica dust put on TB preventive treatment during the reporting period
Proportion of patients with silicosis referred for additional diagnostic tests if NAAT is negative but chest X-ray is positive	Number of patients with silicosis referred for additional diagnostic tests (e.g., HRCT, bronchoalveolar lavage, cultures) during the reporting period	Number of patients with silicosis with NAAT negative but chest X-ray positive results during the reporting period

### Supervision and coordination

Ensure sufficient human resources, training, logistics, and budgetary approvals for successful implementation of bi-directional TB-silicosis activities. Regular review and coordination meetings between stakeholders should be scheduled, with joint monitoring visits to assess staff comprehension, treatment quality, and referrals.

### Private sector involvement

Engage private practitioners, especially pulmonary medicine experts, in the joint TB-silicosis initiative. Encourage private practitioners to collect occupational history from TB patients and maintain high clinical suspicion for silicosis. Non-governmental organizations should be encouraged to refer suspected cases of silicosis to government health clinics for TB testing.

### Implementation plan

Adopt the collaborative TB-silicosis framework in stages, starting with demonstration districts, and scale up based on evidence of success. Use insights from current TB-comorbidity initiatives to guide implementation, focusing on training, action plan preparation, and coordinated efforts across national and state levels (refer to Table 4 for detailed responsibilities and activities).

**Table 4:** Implementation plan for collaborative TB-silicosis activities in India

Activities	Responsibility
**Policy and direction**	
Issue directions to state-level stakeholders/program managers for TB-silicosis joint activities	National nodal officers from the Ministry of Labor and Ministry of Health & Family Welfare
Finalize state action plan for TB-silicosis joint initiative, including required revisions; establish state-level committees for oversight	State nodal officers, state-level program managers, program officers, consultants, silicosis experts, TB-comorbidity committee
**Training and sensitization**	
Conduct cascade training, starting with district-level program managers and nodal officers (including silicosis counseling)	State nodal officers, state-level program managers, program officers, consultants
Train and sensitize staff working in the national TB program and government health centers	District-level nodal officers, district-level program managers, program officers, consultants
Incorporate frontline worker training/sensitization in other national health initiatives with weekly/monthly review sessions	Primary care physicians
**Implementation and monitoring**	
Implement joint TB-silicosis activities	TB health workers, counselors, government health center staff
Conduct collaborative visits for supportive supervision and monitoring	District-level program managers, program officers, consultants
**Stakeholder roles and responsibilities**	
Monitor and control silica dust levels	Safety/ environment/ hygiene divisions in factories/ mines/ ports/ building and other construction work/ plantation work
Conduct periodic medical examinations for occupational diseases, including silicosis	Factory medical officers (FMOs), Medical officers (MOs) at mines/ ports/ building and other construction work/ plantation work
Ensure reporting and documentation of silicosis cases	FMOs, Occupier (owner of factories), Chief medical & health officers (CMHOs), ESIC
Facilitate TB testing for workers diagnosed with silicosis	FMOs, MOs at mines/ports, Diagnosing physicians/ pulmonologists/ radiologists, CMHOs, medical officers of ESIC
Facilitate silicosis diagnosis among TB patients exposed to silica dust	CMHOs, District tuberculosis officers (DTOs), TB health workers, government health center staff, Occupational Disease Center (ODC) at ESIC medical college and hospitals
Supervise the implementation of TB-silicosis activities and ensure compliance with guidelines	Chief Inspector of Factories/ Directorate of Industrial Safety and Health (DISH) of respective state, Directorate General of Factory Advice Service & Labour Institutes (DGFASLI), Directorate General of Mines Safety (DGMS), Central tuberculosis division (CTD), State tuberculosis officers (STOs)
Coordinate and oversee national TB-silicosis joint initiatives	National Tuberculosis Elimination Program (NTEP) officials, CTD, STOs, WHO-NTEP, International Labour Organization (ILO)
Provide technical support and resources for TB-silicosis initiatives	National Institute of Occupational Health (NIOH), DGFASLI
Promote awareness and preventive measures for silicosis and TB among workers	DGFASLI, DGMS, FMOs, MOs, CMHOs, DTOs, NIOH, TB health workers, government health center staff
Develop and enforce regulations related to occupational health and safety	Ministry of Labour & Employment, Ministry of Health & Family Welfare

## Implications

The proposed collaborative TB-silicosis framework has significant implications for public health in India, particularly in regions with high occupational exposure to silica dust. Successful implementation requires coordinated efforts among key stakeholders, including the ministries of health, labor, industry, and environment. The following recommendations outline concrete steps for integrating joint TB-silicosis activities into existing healthcare programs.

### Upgrading healthcare infrastructure

To support early diagnosis and treatment of both tuberculosis (TB) and silicosis, block-level community health centers (CHCs) must be upgraded with essential facilities, including in-patient care, X-rays, and pulmonary function testing. In India, a block is an administrative division typically consisting of a group of villages or towns, serving as a local unit for government administration and public services. Upgrading healthcare infrastructure at this level is crucial, especially in areas near silica-dust-producing industries. Block-level CHCs should be equipped with digital X-ray systems and the necessary software for reading and interpreting digital chest X-rays according to ILO criteria for pneumoconiosis. This includes ensuring the quality of the imaging equipment, adherence to a protocol for obtaining and processing images, and quality control guidelines to avoid issues in classification. Additionally, the facilities should have systems for secure storage and electronic transmission of digital X-rays. Further, training healthcare personnel in using this technology and interpreting the images is essential. Establishing strong referral systems from CHCs to district-level tertiary care centers will ensure continuity of care for patients requiring more advanced treatment.

### Cost reduction for diagnostic services

To reduce the financial burden on workers in silica-prone industries, the government should explore partnerships with private providers to subsidize the costs of X-rays and high-resolution computed tomography (HRCT). Utilizing funds from patient welfare committees can further support low-income patients in accessing these services. Such measures would ensure that financial constraints do not hinder early diagnosis and effective treatment.

### Workplace-based interventions

Workplace-based interventions offer an effective approach to early diagnosis. Deploying mobile digital X-ray vans can facilitate on-site silicosis screening, while sputum samples for TB testing can be collected at the workplace and sent to government health centers for nucleic acid amplification testing (NAAT). Employers can play a pivotal role as treatment supporters, encouraging workers diagnosed with TB to complete their treatment.

### Limitations and future directions

While the expert panel approach provides valuable insights, potential biases due to limited representation must be acknowledged. Ongoing monitoring and evaluation of the collaborative framework will be crucial to ensure its effectiveness and adapt to emerging challenges. The role of the expert panel extends beyond providing recommendations; it also involves advocating for policy changes. By engaging with key stakeholders, including government officials, healthcare administrators, and policymakers, the panel can drive the adoption of its recommendations into official policies. This process typically involves presenting evidence-based findings, participating in policy discussions, and leveraging existing networks within the healthcare and governmental systems. Once adopted, these policies can significantly impact public health outcomes by standardizing practices, securing funding, and ensuring the widespread implementation of the collaborative framework. Future research should focus on understanding the long-term impact of the collaborative framework and exploring innovative approaches to address any emerging challenges.

## Conclusion

Early detection of TB is critical for controlling the spread of the infection, while early diagnosis of silicosis can help workers avoid further exposure to harmful silica dust. The collaborative framework for joint TB-silicosis activities offers a practical approach to achieving these objectives, supporting the early diagnosis and management of two diseases targeted for global elimination by 2030. By integrating TB and silicosis programs, the proposed framework can improve patient outcomes, enhance public health, and contribute to India's national goals for TB elimination. This collaborative approach underscores the need for cooperation among various stakeholders, including government ministries, healthcare providers, and industry representatives, to ensure successful implementation. The framework presented in this policy document outlines the key components for implementing bi-directional collaborative TB-silicosis activities in India. It provides a roadmap for early detection, comprehensive management, and coordination across healthcare systems. The proposed collaborative activities can significantly impact public health, reduce the burden of these diseases, and create a healthier work environment.

## Data Availability

Not applicable.

## Abbreviations

CHC: Community Health Center
DALY: Disability Adjusted Life Years
ESIC: Employees State Insurance Corporation
HIV: Human Immunodeficiency Virus
HRCT: High Resolution Computed Tomography
IEC: Information Education and Communication
NAAT: Nucleic Acid Amplification Test
NTEP: National Tuberculosis Elimination Program
PHI: Peripheral Health Institutions
TB: Tuberculosis
TPT: Tuberculosis Preventive Treatment
WHO: World Health Organization
